# Multi-View Fusion-Based 3D Object Detection for Robot Indoor Scene Perception

**DOI:** 10.3390/s19194092

**Published:** 2019-09-21

**Authors:** Li Wang, Ruifeng Li, Jingwen Sun, Xingxing Liu, Lijun Zhao, Hock Soon Seah, Chee Kwang Quah, Budianto Tandianus

**Affiliations:** 1 State Key Laboratory of Robotics and System, Harbin Institute of Technology, Harbin 150001, China; 15b908017@hit.edu.cn (L.W.); 18S008061@stu.hit.edu.cn (J.S.); 2 School of Physical Sciences, University of Science and Technology of China, Hefei 230026, China; lx2014@mail.ustc.edu.cn; 3 School of Computer Science and Engineering, Nanyang Technological University, Singapore 639798, Singapore; ashsseah@ntu.edu.sg (H.S.S.); btandianus@ntu.edu.sg (B.T.); 4 ST Electronics (Training & Simulation Systems) Pte Ltd., Singapore 567714, Singapore; quah.cheekwang@stee.stengg.com

**Keywords:** 3D object detection, multi-view fusion, semantic segmentation, Manhattan frame

## Abstract

To autonomously move and operate objects in cluttered indoor environments, a service robot requires the ability of 3D scene perception. Though 3D object detection can provide an object-level environmental description to fill this gap, a robot always encounters incomplete object observation, recurring detections of the same object, error in detection, or intersection between objects when conducting detection continuously in a cluttered room. To solve these problems, we propose a two-stage 3D object detection algorithm which is to fuse multiple views of 3D object point clouds in the first stage and to eliminate unreasonable and intersection detections in the second stage. For each view, the robot performs a 2D object semantic segmentation and obtains 3D object point clouds. Then, an unsupervised segmentation method called Locally Convex Connected Patches (LCCP) is utilized to segment the object accurately from the background. Subsequently, the Manhattan Frame estimation is implemented to calculate the main orientation of the object and subsequently, the 3D object bounding box can be obtained. To deal with the detected objects in multiple views, we construct an object database and propose an object fusion criterion to maintain it automatically. Thus, the same object observed in multi-view is fused together and a more accurate bounding box can be calculated. Finally, we propose an object filtering approach based on prior knowledge to remove incorrect and intersecting objects in the object dataset. Experiments are carried out on both SceneNN dataset and a real indoor environment to verify the stability and accuracy of 3D semantic segmentation and bounding box detection of the object with multi-view fusion.

## 1. Introduction

In an indoor environment, objects are regarded as the main contents and they provide crucial clues for scene understanding and environmental perception. Object detection helps the indoor service robot possess higher semantic awareness of its operating environment. As the robot operates in a 3D environment, 3D object detection in a cluttered indoor scene is an essential module for the perception and operation of a service robot, and even for better natural human-robot interaction.

Object detection is one of the fundamental problems in the field of computer vision. It classifies each object in an image and labels the object position using a 2D rectangular bounding box. With the advancement of deep learning technology, image-based object detection algorithms, such as Faster R-CNN [1], SSD [2], YOLO [3,4], and Mask R-CNN [5], have achieved extremely high accuracy. However, these methods rely on training datasets. Mask R-CNN is one of the best performing algorithms in 2D semantic segmentation, but it still shows poor performance in some real indoor environments such as inaccurate semantic masks or error labels. The robustness and accuracy of these methods are still not enough for a robot to operate in 3D space. In addition, it is also difficult to detect an object’s actual position in a 3D environment and to obtain the object’s occupied volume from an object detected in a 2D image. Therefore, it is not robust enough to be used by a robot to perform tasks such as obstacle avoidance navigation or essential object grabbing. To resolve this problem, 3D object detection emerges as a candidate to realize object classification and detection with the position and volume information.

**Figure 1:**
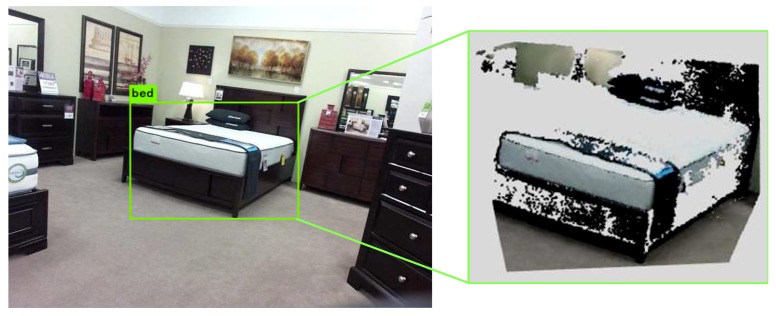
Object point cloud extraction of an image from the SUN RGB-D dataset. Object detection is conducted by YOLO V3 and an object point cloud is extracted by utilizing RGB and depth images. There are many points missing in the object point cloud.

With the invention of depth cameras such as the Kinect sensor, the object’s image and depth information can be easily retrieved. 3D object detection based on the RGB-D information becomes mainstream research. There are some open-source datasets such as NYU V2 [6] and SUN RGB-D [7] which provide a large number of color and depth images with object labels and 3D bounding boxes. In order to compute 3D object bounding boxes, the latest 3D object detection algorithms leverage neural networks to train on datasets, such as DSS [8], PointNet [9,10], and Frustum PointNet [11]. At present, the network structure of the 3D detection algorithm based on the convolutional neural network is still quite complex. Such an algorithm requires a quite high-performance equipment to perform training and forward inference; moreover, it is too time-consuming to deploy it in a robot. In addition, the accuracy of 3D object detection depends on the object amount and categories in a training dataset. Furthermore, most methods utilize a single RGB-D training image of the same object in the NYU V2 and SUN RGB-D datasets. Therefore, the object depth information from only one viewpoint is used, which lead to inaccurate information due to noise and missing information due to occlusion. Hence, 3D object detection using a point cloud faces great challenges because of the fragmented information. Figure 1 is an example from SUN RGB-D dataset which shows a lot of points missing in the object point cloud. In this paper, we explore the multi-view fusion method to resolve the incomplete point cloud information of objects.

Though these datasets provide many RGB and depth images to train, the situation is different when an actual robot operates in a room. As the robot can move freely in an indoor room, the same object may be detected many times in different keyframes. Therefore, we have to deal with the multiple detections of the same object to ensure the uniqueness of the object. Moreover, sometimes the same object observed in multiple viewpoints may be detected with different labels due to inaccurate detection. For example, a chair is detected as a sofa. Another case is that a part of the object may be detected as other label and this leads to an intersection between objects in the same space. Sometimes objects are detected to be very small or large compared to the actual object size. Thus, there are still many problems for a robot to perform 3D object detection in an actual environment.

To resolve these problems, we propose a 3D object detection framework which fuses multiple views and maintains an object database automatically. We leverage the multi-view fusion method to achieve the more complete observation of objects when the robot moves. Then, this will benefit the accurate 3D size estimation of objects. Since the object detection algorithm based on the 2D image has a high recognition precision in some situations, this algorithm can be utilized to detect the object in a 2D image before the object point cloud is segmented. Through the movement of an indoor service robot, images of the same object from different viewpoints can be conveniently obtained, and its pose in each keyframe is estimated using a visual SLAM (Simultaneous Localization and Mapping) algorithm. Therefore, multiple point clouds of the same object can be fused together as the input data of 3D object recognition and it can solve the problem caused by the single object’s incomplete point cloud. The main contributions of this paper are as follows:

(1) We propose a two-stage 3D object detection framework by fusing multiple views of a 3D point cloud based on a real-time visual SLAM for an indoor service robot. Keyframes are continuously processed and 3D bounding boxes of objects are estimated during the motion of a robot.
(2) We construct an object database and propose an object fusion criterion to maintain it automatically. We also propose an object filtering approach based on prior knowledge including size and volume ratio to remove atypical (based on object dimension) and intersecting objects in the object dataset.

The rest of the paper is organized as follows: Section 2 discusses related work on 3D object detection. Section 3 describes the details of 3D object detection utilizing multi-view. Experiments are conducted in Section 4. Conclusions and some suggestions for future work are given in Section 5.

## 2. Related Work

With the use of increasing number of service robots in indoor environments, there is a great deal of work to study the perception of the environment where the robot is operating. For example, the well-known SLAM problem is real-time pose estimation and mapping. In this field, there are a large number of achievements, such as laser-based methods, gmapping [12], Cartographer [13] and vision-based methods, ORB-SLAM2 [14], ElasticFusion [15] and so on, but these are more concerned with the geometric properties of the environment which are applied to the robot to perform tasks such as motion planning and navigation. However, the interaction in a higher level between the robot and the environment (such as semantic interaction and grabbing objects) requires the ability to sense objects in the environment including identifying objects’ categories and obtaining the occupied space of the objects.

Numerous works [16,17,18], have studied semantic segmentation based on RGB and depth images, but the purpose of these works is only to better understand the images in 2D. They do not identify 3D bounding boxes around the detected objects. Lin et al. [19] were the first to propose a method to generate 3D bounding boxes by utilizing the Constrained Parametric Min-Cuts (CPMC) algorithm with 2D appearance features, object-object, and object-scene context relationships are incorporated into a Conditional Random Field (CRF) model for semantic label inference.

Many 3D object detection works have been inspired by the successful application of convolutional neural networks in the field of 2D object detection. Song et al. [8] presented a 3D ConvNet approach which applied ConvNets for 3D object detection. The network includes two main modules which are named Region Proposal Network (RPN) and Object Recognition Network (ORN), respectively. The object proposal method is very similar to the method of 2D object detection. As the two networks do not share layers, the number of parameters is very large. Therefore, the ConvNet method is very time-consuming, requiring about 20 seconds per image.

Deng et al. [20] designed a convolutional neural network that directly leveraged 2.5D (RGB and depth image) images as network inputs to regress the object’s 3D bounding box. The object category detected from an RGB image and the object volume from a depth image are used as the initial values of the network. However, it also has the problem of a large amount of network structure parameters and high operational requirements.

Ren and Sudderth [21] proposed a cloud of oriented gradients descriptor for classifying 3D bounding boxes. Contextual features are fused in this method. Although it achieves high accuracy on the SUN-RGBD dataset, it needs a long time to compute features. Lahoud et al. [22] presented a 3D object detection method for orientating, placing, and scoring bounding boxes around objects by utilizing 2D information in order to quickly reduce 3D search space. 2D object detection is employed to detect objects and their locations and sizes are learned by a Multilayer Perceptron (MLP). In the final step, they refine the detections based on the relations of object categories in a scene. Nevertheless, object point cloud from 2D object detection inadvertently includes some background points which could result in an inaccurate estimation of orientation.

In most of these methods, only a single RGB or RGB-D image is utilized to estimate the 3D bounding boxes of objects. However, this cannot obtain a complete object point cloud information from a single perspective. This seriously affects the object detection. In this paper, we solve this problem by fusing multiple keyframes which are obtained in a real-time visual SLAM. Moreover, unlike the previous method [8] that assumes all objects in the scene shared the same orientation, we estimate the orientation for each object. Compared with Lahoud et al.’s approach [22], we remove the wrong background points from the detected object point cloud by utilizing an unsupervised point cloud segmentation algorithm which can obtain a more accurate orientation estimation.

Multi-view methods have been developed in other areas such as semantic segmentation. Antonello et al. [23] proposed a multi-view frame fusion method to improve the semantic segmentation and to create accurate semantic maps. Tateno et al. [24] studied object recognition on 3D data which was reconstructed by multi-view frames in order to improve the robustness. They propose a framework to carry out incremental segmentation of a 3D scene. Nakajima et al. [25] put forward an object-oriented semantic mapping approach based on InfiniTAM [26] which was a visual SLAM method using continuous RGB-D frames. This method also utilizes the 3D dense map to conduct semantic segmentation. Grinvald et al. [27] also focused on object-centric map construction and conducted the object fusion from multiple segmented point clouds. However, 3D object bounding box and object pose, which are very important for robot operation and environmental perception, are not estimated in these methods. In this paper, we fill this gap by providing multiple properties of 3D objects. Moreover, the error detection cases and the intersection of object bounding boxes are rarely involved in other studies. We will study these in the situations in which a robot may encounter an actual environment.

The 3D object detection for a service robot requires object point cloud integrity, unique labeling, and accurate 3D bounding box. In this paper, we propose a framework to perform 3D object detection continuously, to construct an object database, and to maintain it automatically.

## 3. 3D Object Detection Algorithm Based on Multi-View Fusion

**Figure 2:**
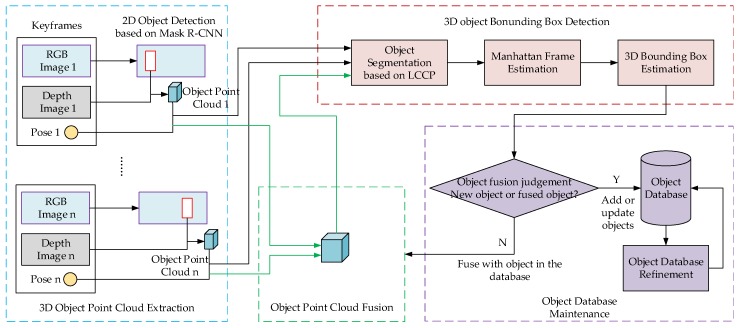
Schematic diagram of the 3D object detection algorithm based on multiple keyframes fusion observation.

A service robot usually employs an RGB-D camera as the main visual sensor in order to perceive an environment. We utilize the RGB and depth images to perform 3D object detection in the environment in order to obtain the object category and 3D bounding box. This section introduces the proposed algorithm of 3D object detection based on multiple views fusion (schematic diagram is shown in Figure 2). It includes five modules:

1. Object point clouds extraction based on Mask R-CNN;
2. Unsupervised segmentation of the object point cloud;
3. 3D object bounding box estimation based on Manhattan Frame;
4. Object point cloud fusion utilizing multi-view and object database maintenance;
5. Object database refinement to remove error detection and object intersections.

### 3.1. Object Point Cloud Extraction Based on Mask R-CNN

This module detects and extracts object point clouds in each input image. We utilize a visual SLAM method to generate keyframes and detect objects based on a deep learning method. Finally, object point clouds are extracted and transformed into the world coordinate system using the pose of each keyframe. As a service robot moves, continuous pose estimation is always conducted in order to obtain its pose in the world coordinate system. In this paper, the visual SLAM algorithm named ORB-SLAM2 [14] is employed as it can achieve high accuracy and robustness when using an RGB-D camera in an indoor environment. ORB feature points are extracted and matched among input frames, and the frame pose is calculated. To balance the performance and efficiency, the frame with enough inliers and low overlap from other frames are selected and named as a keyframe. The rigid 3D pose of keyframe *n* is a 4 × 4 transformation matrix $T_{n}=[R_{n},t_{n}]\in \mathrm{SE}(3)$ which consists of a rotation matrix $R_{n}\in \mathrm{SO}(3)$ and a 3D translation vector $t_{n}\in R^{3}$.

Inspired by the effectiveness of object instance segmentation in 2D images based on deep learning, we employ Mask R-CNN to detect objects, which is an end-to-end method to extend the Faster R-CNN method by adding a branch to predict instance segmentation masks. It can perform quite well in the test dataset (at 5fps in a GPU Pascal Titan X, the instance segmentation accuracy of 35.7% mAP using ResNet-101-FPN as the backbone in the Microsoft COCO dataset). To improve the processing efficiency, object detection is only conducted on each keyframe. For the *n*-th keyframe (n∈R), we can obtain several detected objects $\varphi_{k}^{n}(k\in [1,m])$ (*m* is the number of objects) which includes some properties such as class ID $C_{k}^{n}\in \{0,1,\cdots ,80\}$, probability $\psi_{k}^{n}\in [0,1]$, bounding box $B_{k}^{n}$, and object instance mask $M_{k}^{n}$ which is a binary image with the same resolution as the RGB image.

We set a threshold λ of the object probability, then only the object that satisfied $\psi_{k}^{n}\geq \lambda$ is selected to extract point cloud. We can extract object point cloud ${}^{C}P_{k}^{n}(k\in [1,m^{\prime }])$ in the scope of detected object instance mask $M_{k}^{n}$ using the RGB and depth images ($m^{\prime }$ is the number of objects meeting the threshold condition, and the left superscript C means the current coordinate system {*O_C_*} of the keyframe). For the convenience to describe objects, object point cloud ${}^{C}P_{k}^{n}$ should be transformed into the world coordinate system {*O_W_*}. We leverage the *n*-th keyframe’s pose transformation matrix $T_{n}$ to convert, and then obtain the object point cloud $P_{k}^{n}$ in the world coordinate system (for convenience, if we do not declare the coordinate system, it is in the world one):

(1) $$P_{k}^{n}=T_{n}{}^{C}P_{k}^{n}$$

### 3.2. Unsupervised Segmentation of the Object Point Cloud

Object point cloud detection using 2D image usually contains background point cloud (in addition to the object point cloud) which greatly affects the estimation accuracy of the 3D object bounding box. Therefore, we need to extract the object point cloud from the background point cloud.

In order to segment the object, the geometric unsupervised learning method called Locally Convex Connected Patches (LCCP) is leveraged. Before segmentation, the point cloud is processed by a voxel filter, a pass-through filter, and a statistical filter to remove noise points. Then, the super-voxel segmentation is conducted that the point cloud is subdivided into many small blocks according to spatial position and point cloud surface normal vector. The adjacency graph is later computed to connect nearby super voxels. Finally, super voxel clustering is performed by utilizing convexity and concaveness between small blocks. The angle between the centerline vector of adjacent super voxel block and the normal vector is calculated to confirm the convex-concave relationship. After marking the convex-concave relationship of each block, the region growth algorithm that the region is only allowed to grow across the convex side is adopted to cluster smaller super blocks into larger objects. Since the point cloud density decreases with the distance increases, it is difficult to determine the octree size. Thus, logarithmic transformation is applied in the z-axis orientation in order to improve the accuracy.

After the above operation, the point cloud is segmented into s regions. A region in which the object belongs to needs to be extracted. Since the object is usually at the center of the bounding box and occupies most of the area in the box, the segmented point cloud ${}_{seg}P_{k}^{n}$ of an object $P_{k}^{n}$ can be selected according to the maximum number of points among all segmented point clouds:

(2) $${}_{seg}P_{k}^{n}=\underset{{}_{seg}P_{kv}^{n}}{\arg\max}(\langle {}_{seg}P_{kv}^{n}\rangle )(v\in [1,s])$$

where $\langle {}_{seg}P_{kt}^{n}\rangle$ means the number of points in the *v*-th segmented point cloud ${}_{seg}P_{kv}^{n}$.

In Nakajima and Saito’s method [25], the object segmentation is always done in the whole image point cloud. However, we only conduct the segmentation in the object point cloud in order to reduce the calculation.

### 3.3. 3D Object Bounding Box Estimation Based on Manhattan Frame

After the object ${}_{seg}P_{k}^{n}$ is segmented from the point cloud, its 3D bounding box will be estimated. Since the object point cloud coordinates are in the world coordinate system {*O_W_*}, a 3D bounding box can be obtained by directly calculating the maximum and minimum values of the object point cloud in three axial orientations. However, when the object is in different positions, it might obtain 3D bounding boxes with different sizes, which cannot effectively determine the spatial volume occupied by the object.

Generally, objects in a man-made environment often contain orthogonal or parallel planes, and the Manhattan world assumes that each plane is perpendicular to an axis in a coordinate system (Manhattan World Assumption). Such a coordinate system is called Manhattan Frame (MF), which characterizes the main direction of object distribution. Therefore, MF can be utilized to solve the object orientation before calculating the 3D bounding box.

The rotation matrix R of Manhattan Frame is calculated by utilizing the algorithm proposed in [28]. We calculate the surface normal $\mathbb{N}={\left\{{\vec{n}}_{i}\right\}}_{i=1}^{N}(N\in R)$ of an object point cloud and regard the normal as the input to estimate MF. Then, we consider an MF estimation problem as a consensus set maximization that maximizes the number of inliers over the rotation search space:

(3) $$\underset{R\in SO(3)}{\arg\max}\sum_{i=1}^{N}\sum_{j=1}^{6}〚∠\left({\vec{n}}_{i},R{\vec{e}}_{j}\right)\leq \delta 〛$$

where $∠\left({\vec{n}}_{i},R{\vec{e}}_{j}\right)$ is the vector angle between ${\vec{n}}_{i}$ and $R{\vec{e}}_{j}$, ${\left\{{\vec{e}}_{j}\right\}}_{j=1}^{6}$ are the basis vectors and their opposite vectors ${\vec{e}}_{1}{=[1,0,0]}^{T}$, ${\vec{e}}_{2}{=[0,1,0]}^{T}$, ${\vec{e}}_{3}=[0,0,\;{1]}^{T}$, ${\vec{e}}_{4}=-{\vec{e}}_{1}$, ${\vec{e}}_{5}=-{\vec{e}}_{2}$, ${\vec{e}}_{6}=-{\vec{e}}_{3}$, δ is the threshold value for judging inlier points, and 〚⋅〛 is an indicator function.

As the orientations of 3D bounding boxes of most objects in an indoor environment are along the normal direction of the floor, we modify the estimated MF to satisfy this restriction. Then, the rotation matrix R of MF is converted to $R^{\prime }$. After solving the MF coordinate system {*O_MF_*}, the center of object point cloud $p_{c}=(x_{c},y_{c},z_{c})$ is calculated in the coordinate system {*O_W_*}. The coordinate system {*O_MF_*} will be translated to the object’s center, then $\left\{O_{MF}\}=\{R^{\prime },p_{c}\right\}$, and the transformation matrix of the coordinate system from {*O_MF_*} to {*O_W_*} is expressed as TWMF. According to Equation (4), the object point cloud ${}_{seg}P_{k}^{n}$ in the system {*O_W_*} is converted to {*O_MF_*}, expressed as PsegMFkn:

(4) PsegMFkn=TWMF−1⋅segPkn

At this time, the maximum and minimum values of the object point cloud ${}_{seg}P_{k}^{n}$ in three axial orientations are calculated in the coordinate system {*O_MF_*}, thereby we obtain the 3D bounding box ${}^{3D}B_{k}^{n}$ of the object.

### 3.4. Object Point Cloud Fusion Utilizing Multi-View and Object Database Maintenance

The robot leverages a visual SLAM algorithm to generate multiple keyframes when moving. The same object may be observed in multiple views, therefore, fusing point clouds of the same object in multiple views can result in a more complete object. 

**Figure 3:**
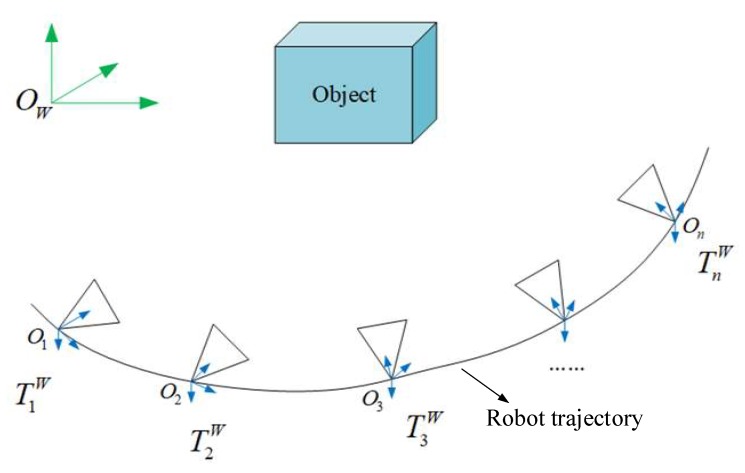
Schematic diagram of the observed object from different views.

A schematic diagram showing an observation of the same object from different viewpoints is shown in Figure 3. We regard each keyframe from the visual SLAM as a new view. The world coordinate is labeled as {*O_W_*}, and the *n*-th keyframe coordinate system is labeled as {*O_n_*} in the visual SLAM. We follow the same method described in Section 3.1 to convert the object point cloud ${}^{C}P_{k}^{n}$ in the *n*-th keyframe to the world coordinate system {*O_W_*}, and then we can obtain $P_{k}^{n}$. 

In each keyframe, several objects can be detected and there may exist multiple objects in the same category. Therefore, it will be a problem to fuse object point clouds among different keyframes. To this end, we design an object database to maintain objects from multiple keyframes. The object database is in the world coordinate system {*O_W_*}, and it includes some properties: object class ID, probability, object ID, object point cloud, segmented object point cloud, 3D bounding box. In order to manage the object database automatically, we design some rules for object addition and update.

#### 3.4.1. The First Time to Insert Objects to the Object Database

Initially, the object database is empty. We detect objects from the first keyframe (n=1) and obtain several detected objects $\varphi_{k}^{n}(k\in [1,m])$ (*m* is the number of objects). All objects in this keyframe will be added to the database only when they satisfy the constraint:

(5) $$\{\begin{array}{c} m\geq 1\\ \psi_{k}^{n}\geq \lambda (n\in N^{+},k\in [1,m]) \end{array}$$

If objects $\varphi_{k}^{1}$ in the first keyframe fail to meet Equation (5), we conduct the same operation in the next keyframe until that it appears the keyframe to meet the condition. We do these because objects with the same category should not fuse together in this keyframe and the object point cloud fusion will be conducted after this keyframe. 

Then, we calculate the properties of objects according to Section 3.1, Section 3.2 and Section 3.3 and add them to the database ℚ. The properties of the α-th object ${\mathbb{Q}}_{\alpha }$ include object class ID $C_{\alpha }$, probability $\psi_{\alpha }$, object ID α, object point cloud $P_{\alpha }$, segmented object point cloud Psegα, 3D bounding box ${}^{3D}B_{\alpha }$ ($\alpha \in [1,\xi ],\;\xi \in N^{+}$, ξ is the number of objects in the database ℚ).

#### 3.4.2. Object Fusion Criterion and Database Maintenance

After the first time we insert objects to the database ℚ, the detected objects in the subsequent keyframes are conducted the object fusion criterion to determine whether to add to the database directly or to fuse with existing objects.

For each detected object $\varphi_{k}^{n+1}$ satisfying Equation (5) in the (*n* + 1)-th keyframe, we search the database ℚ based on object class ID $C_{k}^{n+1}$. We can obtain all objects with the same $C_{k}^{n+1}$ in the database and the number of objects is η(η≤ξ,η∈N). If the value η is zero, it means the object is a new one and can be inserted to ℚ directly. The object ID α is updated by α←α+1. Otherwise, all objects with the same $C_{k}^{n+1}$ are selected to determine which one to fuse. We judge whether it is the same object based on the centroid distance between two 3D object bounding boxes. For the *i*-th object $\left(i\leq \eta ,i\in N^{+}\right)$, the distance $d_{i}$ can be calculated. For each object class, we can set the distance threshold based on the object’s prior size. Finally, we decide to fuse or to insert the object by using Equation (6):

(6) $$\{\begin{array}{c} \min(d_{i})\leq \Gamma_{C_{k}^{n+1}},fuse\\ \min(d_{i})>\Gamma_{C_{k}^{n+1}},insert \end{array}$$

where $\Gamma_{C_{k}^{n+1}}$ is the threshold of the object with the class ID $C_{k}^{n+1}$.

In order to improve the fusion effectiveness, we utilize the original object point cloud $P_{j}^{n+1}$ instead of the segmented one Psegjn+1 to fuse (j is the object ID in the database corresponding to $\min(d_{i})$). And the *j*-th object point cloud is replaced by a fused point cloud:

(7) $$P_{j}^{n+1}\leftarrow P_{k}^{n+1}+P_{j}^{n+1}$$

Subsequently, the fused object point cloud undergoes point cloud filter, segmentation, and 3D bounding box estimation. As an object is observed at different views, the probability of the object may not be the same. We consider that the mean probability value is more comprehensive to the observed object. Then, the probability $\psi_{j}$ is updated by:

(8) $$\psi_{j}\leftarrow mean(\psi_{k}^{n+1},\psi_{j})$$

Finally, the corresponding object in the database is updated by the new object properties, while the object ID is still the old one. After the fusion operation, we can obtain a more completed object to estimate the 3D bounding box. In this way, new keyframes are continuously processed and 3D bounding boxes of objects are estimated during the motion of a robot. Meanwhile, the object database is maintained automatically.

### 3.5. Object Database Refinement

After the above processing, we can obtain a database with many objects. However, due to the inaccurate semantic segmentation of object instances, we may encounter errors such as incorrect object sizes (too small or too big compared with the general object size) and the intersection of object bounding boxes. To this end, we propose an object filtering approach based on prior knowledge including object size and volume ratio to solve the incorrect and intersection detection problems in the object dataset.

#### 3.5.1. Atypical Object Filtering Based on Prior Knowledge

For general objects in an indoor environment (such as the chair, table, sofa, and bed), their typical sizes are known priorly and they are usually within a certain range or threshold. For example, if we detect a chair’s bounding box with the size of 0.10 m × 0.15 m × 0.05 m, we can judge that this is an error estimation and we delete it from the object database.

Therefore, in order to remove the detected atypical objects in the object dataset, the prior sizes of objects are utilized as the criterion. We compare the object volume with the prior one and also contrast the edges of the bounding box. This is because sometimes the object volume meets the constraint, but one of the object edges is too short. For each object category C∈{0,1,⋯,80}, we define the prior size: length $L_{C}$, width $W_{C}$, and height $H_{C}$. In the object database, the *i*-th object $\varphi_{i}$ has the bounding box $B_{i}$ with the size of length $L_{i}$, width $W_{i}$, and height $H_{i}$. Then, we design the discriminant criteria as:

(9) $$\begin{array}{l} \left(L_{i}\cdot W_{i}\cdot H_{i}\geq \Gamma_{V\min}\cdot L_{C}\cdot W_{C}\cdot H_{C}\right)\cap \\ \left(L_{i}\cdot W_{i}\cdot H_{i}\leq \Gamma_{V\max}\cdot L_{C}\cdot W_{C}\cdot H_{C}\right)\cap \\ \left(\min(L_{i},W_{i},H_{i})\geq \Gamma_{E}\min(L_{C},W_{C},H_{C})\right) \end{array}$$

where $\Gamma_{V\min}$ and $\Gamma_{V\max}$ are minimum and maximum volume limit thresholds of the object, respectively, and $\Gamma_{E}$ is the threshold of the object edge.

If the object can satisfy the constraint in Equation (9), we consider the object size is true positive. Otherwise, the object is atypical and it should be removed from the object database. Therefore, the database can be updated automatically.

#### 3.5.2. Intersection Object Filtering Based on Volume Ratio

During the movement of a robot, multiple keyframes are obtained by visual SLAM and then these are conducted instance semantic segmentation by using Mask R-CNN. The same object can be detected for many times and these detections are fused by using the method explained in Section 3.4. However, if the object is detected with a different label, the fusion algorithm will fail. The ‘new’ object will be added to the database again and its bounding box will have a large intersection with the bounding box of the ‘previous’ one. Worse, some parts of a big object are detected with different labels, and these smaller objects are included in the big object. Therefore, we have to deal with these error cases.

**Figure 4:**
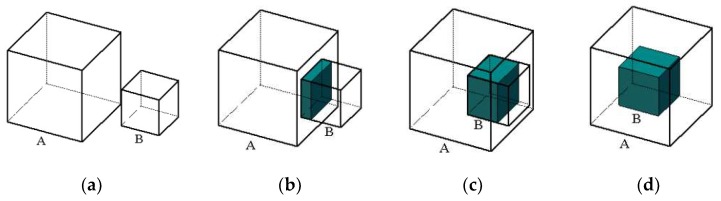
Different situations of object intersection: (**a**) no intersection; (**b**) tiny intersection; (**c**) large intersection; (**d**) fully intersection.

After the analysis of the spatial positional relationship between 3D object bounding boxes, we observe that there are four situations: no intersection, tiny intersection, large intersection, and full intersection, as shown in Figure 4. In order to remove the intersecting object, we design an object filtering algorithm based on the volume ratio to refine the object database. We utilize the volume ratio between the intersection part with the volume of each object in order to decide intersection types. As most objects in the normal indoor environment have Manhattan framework property, we can adjust one axis of Manhattan framework in the 3D bounding box to be parallel to the ground normal vector. We show illustrations of various intersection types between the object A volume $V_{A}$ and object B volume $V_{B}$ in Figure 4.

The volume ratios $\eta_{A}$ and $\eta_{B}$ between the intersection $V_{\cap }$ and the volume of each respective object $V_{A}$ and $V_{B}$ can be calculated by using Equation (10):

(10) $$\{\begin{array}{c} \eta_{A}=V_{\cap }/V_{A}\\ \eta_{B}=V_{\cap }/V_{B} \end{array}$$

We set a minimum volume ratio threshold $\Gamma_{V_{\cap }}$ to decide the intersection type as shown in Equation (11). In Figure 4a,b, the intersection part is small, so we do not delete the objects. In Figure 4c,d, the most part of object B is in inside object A, so we remove object B in the dataset. Therefore, for each object in the database, we compare it with all other objects in order to calculate the volume ratio by using Equation (10). The filtering algorithm is presented in Algorithm 1 (including all cases of $V_{A}\geq V_{B}$ and $V_{A}<V_{B}$):

(11) $$\{\begin{array}{c} (\eta_{A}=0)\cap (\eta_{B}=0),(1)\\ \left(\eta_{A}\leq \Gamma_{V_{\cap }})\cap (\eta_{B}\leq \Gamma_{V_{\cap }}),(2\right)\\ \eta_{B}>\Gamma_{V_{\cap }},(3)\\ \eta_{B}=1,(4) \end{array}$$

**Algorithm 1: Intersection object filtering algorithm**
Ω: the number of objects in the database ℚ D: the array of subscripts that need to be deleted in the database ℚ1: ε=0;2: **for**
σ = 0; σ < Ω; σ++ **do** 3:  **for**
τ = σ + 1; τ< Ω; τ++ **do**4:   Calculate the intersection $V_{\cap }$ between object $\varphi_{\sigma }$ and $\varphi_{\tau }$;5:   $\eta_{\sigma }=V_{\cap }/V_{\sigma }$;6:   $\eta_{\tau }=V_{\cap }/V_{\tau }$;7:  **end for**
8:  $\xi =\underset{\tau }{\arg\max}(\eta_{\sigma +1},\cdots ,\eta_{\tau },\cdots ,\eta_{\Omega })$;9:  **if**
$\left(\eta_{\sigma }>\eta_{\xi })\cap (\eta_{\sigma }>\Gamma_{V_{\cap }}\right)$
**then**10:   D(ε)=σ; 11:   ε++; 12:  **elseif**
$\left(\eta_{\sigma }\leq \eta_{\xi })\cap (\eta_{\xi }>\Gamma_{V_{\cap }}\right)$
**then**
13:   D(ε)=ξ; 14:   ε++;15:  **end if**16: **end for**
17: ε=0;18: **for**
σ = 0; σ < Ω; σ++ **do** 19:  **if**
D(ε)=σ
**then**
20:   Delete the object $\varphi_{\sigma }$ in the database ℚ; 21:  **end if**22: **end for**

## 4. Experimental Evaluation

In order to verify the effectiveness of the proposed multi-view based 3D object detection algorithm, we need to perform experiments on an open-source dataset for quantitative evaluation. Although there are some datasets providing ground truth objects with semantic segmentation and 3D bounding boxes (such as the NYU depth dataset V2, SUN RGB-D), they cannot be used for evaluating our algorithm because these datasets consist of discrete images. Based on this requirement, we select SceneNN [29,30] dataset which provides raw RGB-D data for evaluating our algorithm performance. This dataset is captured by Asus Xtion Pro and Kinect V2 sensors and it consists of more than 100 indoor scenes such as offices, dormitory, classrooms, and so on. All scenes are reconstructed to 3D map representations and have per-vertex and per-pixel annotation. 

To compare our results with previous works [27,31], we ran experiments using those previous works on 10 scenes from SceneNN dataset. In these works, only the object-level 3D semantic segmentation accuracy is evaluated, excluding 3D bounding box accuracy. Therefore, we conduct the object semantic segmentation using our multi-view fusion method in order to compare with these methods. Afterward, the 3D object bounding boxes are given in our method. We provide the details of our experimental results in order to prove the effectiveness of object fusion strategy and object database refinement. Finally, the performance of the method is provided.

### 4.1. Object-Level 3D Semantic Segmentation Evaluation

Many works in 3D object semantic segmentation always take the whole reconstructed scene as the input. These are quite different with our work because there is no fusion problem in multiple partial detections of objects and these methods are not directly applicable for incremental object detection during robot movement. Therefore, we do not compare with these methods in the paper. To the best of our knowledge, the recent works by Pham et al. [31] and Grinvald et al. [27] are most related to our work and we present quantitative results of 3D segmentation accuracy.

In the work by Pham et al. [31], they report 3D object segmentation accuracy for 40 classes in the NYU RGB-D V2 dataset which includes segmentation for non-object classes such as floor, ceiling, and wall. The purpose of this work is to classify every single element in the 3D scene. However, we focus on the object-level 3D detection in the environment for a robot. We adopt the Mask R-CNN which is trained on the Microsoft COCO object dataset with 80 classes. There are nine object categories in common with the NYU dataset. Compared with the work by Pham et al. [31], the work by Grinvald et al. [27] is more similar to our work. Therefore, we follow the test configuration of Grinvald et al. [27] in order to conduct the experiment on the same 10 sequences of SceneNN dataset. We also list the results of the work by Pham et al. [31], which is evaluated in a bigger set of classes.

Our method is evaluated on 10 sequences from the SceneNN dataset. In order to decrease the influence of pose estimation error, the poses provided in the dataset are utilized. Every 10 images of the raw RGB and depth images are regarded as a keyframe input to our method. After object fusion and dataset refinement, we can obtain the point cloud of each detected object. Then, we calculate the object semantic segmentation accuracy by using 3D Intersection over Union (IoU) method. The object ground truth is extracted from the reconstructed triangle meshes with annotation and it is converted to a point cloud. We calculate the 3D IoU between the segmented object points and the one in the ground truth. Finally, we compute the per-class Average Precision (AP) and mean AP on all 10 sequences, as shown in Table 1. In order to illustrate the effectiveness of our method, we compare with the recent previous works [27,31], as shown in Table 2. The results show that our method outperforms Pham et al.’s method [31] on nine of the ten sequences and the Grinvald et al.’s method [31] on six of the ten sequences, respectively, which can prove the object-level semantic segmentation accuracy of our method. In Table 2, we also add a baseline only using single keyframe to verify the fusion strategy of multiple keyframes. The object detection is conducted in each keyframe but the detected objects are not fused together. The results show that the accuracy is very significant decline compared with the fusion one. The cause of this problem is that the object point cloud is incomplete in one frame and this verifies the influence of fusion strategy in our method.

In order to verify the influence of different modules in our method, we carry out the ablation experiments on 10 sequences of SceneNN dataset, as shown in Table 3. Firstly, the point cloud filter and LCCP modules are removed individually to verify the effect. We observe that the results are all reduced and the LCCP module impacts greatly. Then, we remove both two modules. The results become worse compared with previous results, which verifies the effectiveness of each module.

**Figure 5:**
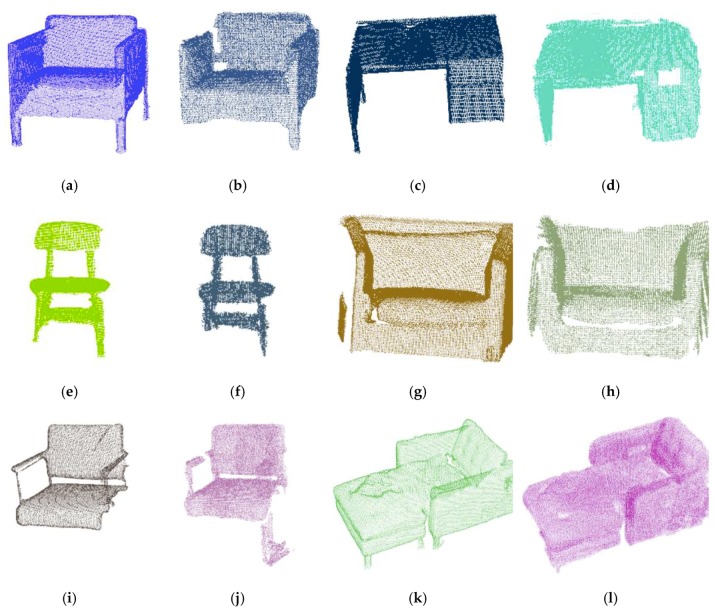
Some 3D object semantic segmentation results compared with ground truth on the SceneNN dataset (the first and third columns including (**a**,**c**,**e**,**g**,**i**,**k**) and are ground truth, the second and fourth columns including (**b**,**d**,**f**,**h**,**j**,**l**) are extracted by our method).

Besides, we also provide several segmented point clouds of objects on these scenes as shown in Figure 5. To compare the segmentation effectiveness, the corresponding ground truths of objects are also given. It can be seen that the object can be segmented accurately.

### 4.2. 3D object Bounding Box Detection and Database Refinement

**Figure 6:**
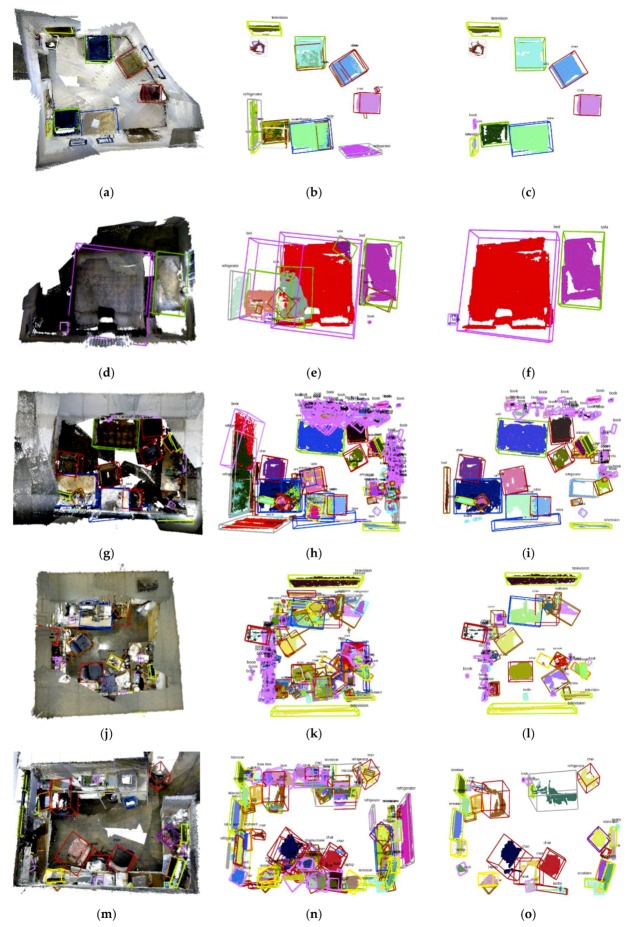
3D object bounding box detection experiments on the SceneNN dataset (from top to down, sequence number: 011, 016, 030, 078, 086; the first column (**a**,**d**,**g**,**j**,**m**) is the final detection with point cloud map, the middle column (**b**,**e**,**h**,**k**,**n**) is the detection objects before database refinement, the final column (**c**,**f**,**i**,**l**,**o**) is the detection objects after database refinement).

In addition to segmenting the object point cloud, we also carry out 3D bounding box detection. We adopt an oriented bounding box which can enclose the object compactly. Manhattan frame method is utilized to estimate the main orientations in three orthogonal axes. One axis of Manhattan framework in each box has been adjusted to be parallel to the ground normal vector. Some experiments on the SceneNN dataset are presented in Figure 6. Through the fusion of multi-view observation and 3D bounding box estimation, objects are detected and added to the database. In order to validate the robustness of our object fusion and database refinement algorithm, we present not only point clouds and 3D bounding boxes in the database, but also detection results before and after database refinement (shown in the middle and right column respectively in Figure 6). We observe that there are a lot of objects with atypical sizes and huge intersection volume before database refinement. Most of these atypical or intersecting objects are removed after the refinement, which can prove the effectiveness of our method.

**Figure 7:**
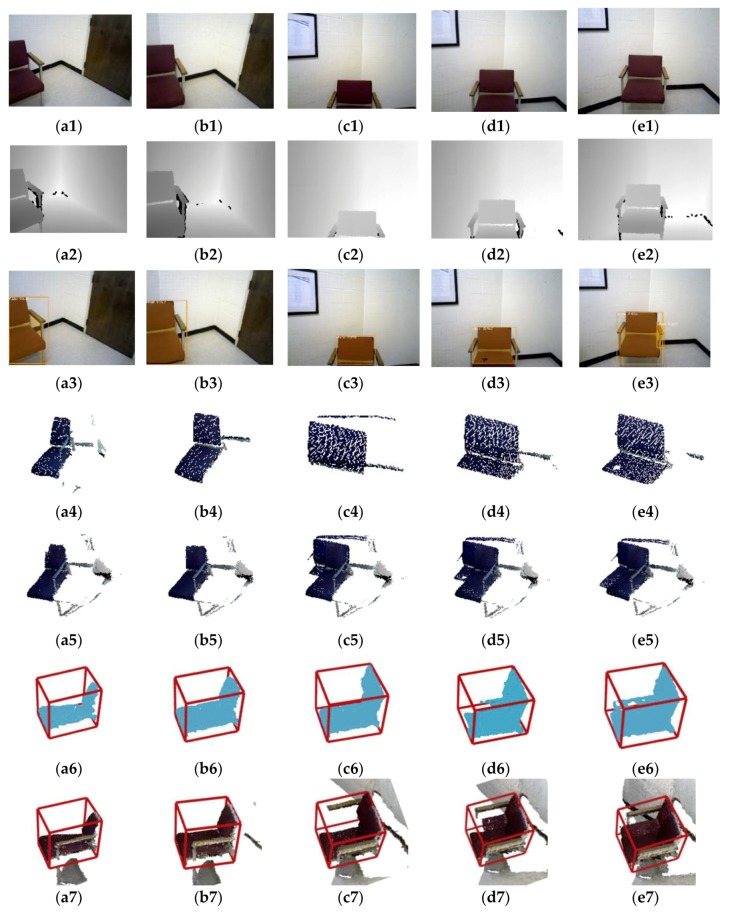
One example of 3D object detection on the SceneNN dataset(sequence ID: 011, (**a1**–**e1**) are the input RGB images as keyframes, (**a2**–**e2**) are depth images, (**a3**–**e3**) are the object instance segmentation by Mask R-CNN, (**a4**–**e4**) are the extracted object point cloud, (**a5**–**e5**) are the fused object point cloud using multi-keyframe, (**a6**–**e6**) are the segmented object point cloud based on LCCP and 3D bounding box based on Manhattan frame estimation, and (**a7**–**e7**) are the final 3D object detection in the point cloud map). The chair can be merged more completely and the 3D bounding box is estimated more accurate after multi-keyframe fusion.

Additionally, one example of object fusion progress is given to show the details, as shown in Figure 7. Multiple views of the object “chair” from the 011 sequence of the SceneNN dataset are detected and fused gradually. The shown progression includes RGB and depth images, detected mask images, extracted object point clouds, fused object point clouds, segmented object point clouds with 3D bounding boxes, and objects in 3D map representation. The object point clouds from different perspectives are extracted and fused together to form a more completed object. However, object point cloud obtained by the Mask R-CNN detection always contains some background points (the third and fifth rows in Figure 7), and this will have a serious impact on 3D bounding box estimation. We adopt the geometric segmentation method based on LCCP in order to remove background points and then estimate the box (the sixth row in Figure 7). The final results verify the effectiveness of our method.

**Figure 8:**
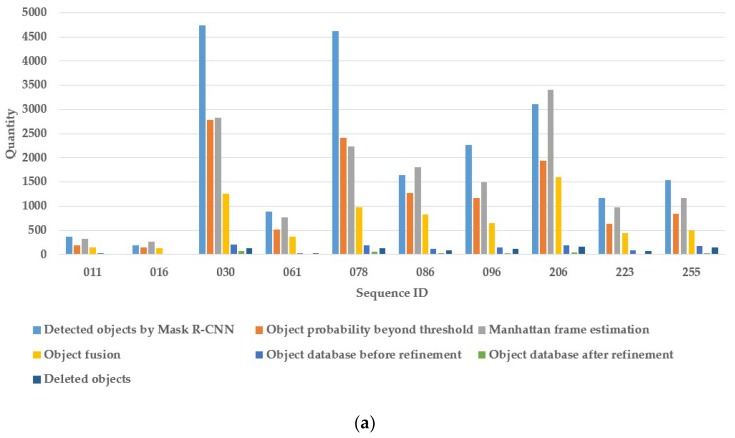

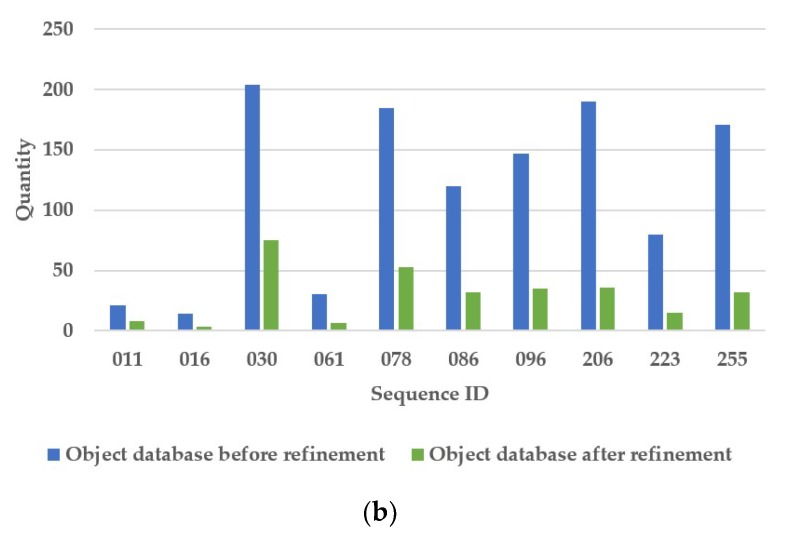
Comparison of the number of database objects before and after refinement processing on the SceneNN dataset: (**a**) 3D object detection column chart; and (**b**) object database column chart.

Furthermore, we present the details of our method results when running the 10 sequences of SceneNN dataset, shown in Table 4. For each sequence, the numbers of images, keyframes, detected objects by Mask R-CNN, Manhattan frame estimation, object fusion, database objects before and after refinement are counted. These data are shown in a column chart in Figure 8a and the numbers of database objects before and after refinement are also given in Figure 8b. We can observe that a large number of same objects are detected in multiple keyframes, and our method can work well to conduct 3D object detection continuously and maintain the object database automatically.

**Figure 9:**
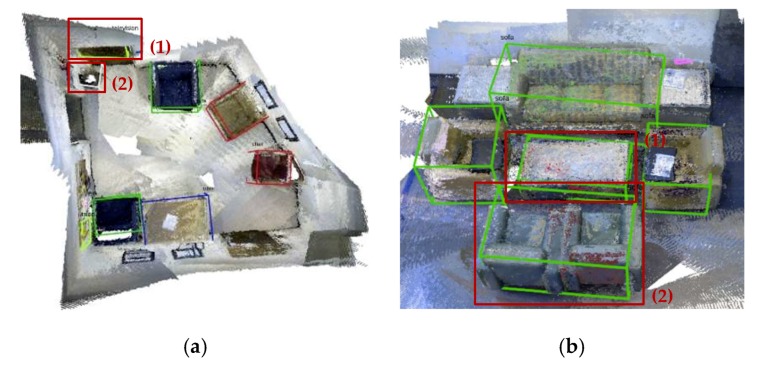
Some failure cases of 3D object detection on the SceneNN datasets. (**a**) sequence 011, (1) the door is recognized as a television and (2) the dustbin is recognized as a toilet; (**b**) sequence 061, (1) the table is recognized as a sofa and the two sofas are recognized as a big sofa.

After the experiments on the SceneNN dataset, we observe that our method can achieve good results for most situations. However, we also discover some failure cases. Several examples are shown in Figure 9. For Figure 9a1,a2,b1 these cases are caused by error category detection of Mask R-CNN. We can improve the performance by fine-tuning Mask R-CNN using indoor environmental images in the future. For Figure 9b2, this case is error fusion of bounding boxes as two sofas are next to each other.

**Figure 10:**
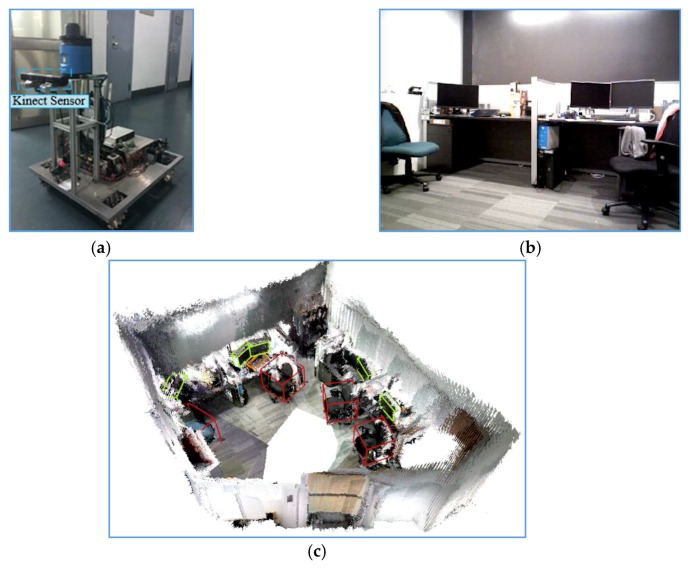
The real environmental experiment using a robot: (**a**) the robot with a Kinect V1 sensor; (**b**) the image of real indoor environment; and (**c**) the 3D object detection results with point cloud.

In addition to the experiments on the SceneNN dataset, we also carried out an experiment in the real indoor environment using a service robot, as shown in Figure 10. The Kinect V1 RGB-D sensor installed in the robot was used to produce RGB and depth images and the ORB-SLAM method is utilized to generate the keyframes and poses. Then, our method was used to detect 3D objects. Finally, 11 objects including four chairs, five televisions and two keyboards are detected, as shown in Figure 10c.

### 4.3. Runtime Evaluation

The proposed method for the 3D object semantic segmentation and detection of a robot is evaluated on a laptop equipped with an Intel i9 CPU operating at 3.30 GHz and an Nvidia Titan RTX GPU. The GPU is used for 2D object detection based on Mask R-CNN. All the experiments are carried out using the RGB-D images with a resolution of 640 × 480 pixels as the input and the point cloud resolution is set to 0.01 m. The average runtime (over 10 sequences of the SceneNN dataset) of the main components of our method is given in Table 5.

Although our method operates at a not very high frequency, it can provide a continuous detection for an indoor robot to obtain object-level environmental perception ability which benefits the robot in a real application.

## 5. Conclusions

This paper proposes a two-stage 3D object detection algorithm to conduct object-level indoor scene perception for robots. The object point clouds from multiple views are fused and 3D bounding boxes are calculated. We construct an object database and propose an object fusion criterion to maintain it automatically. An object filtering approach based on prior sizes and volume ratio is proposed to remove atypical and intersection detections in the object dataset. Experiments carried out on the SceneNN dataset and real environments verify the effectiveness and accuracy of the proposed method. In the future work, the object-level map can be used for the operation of a service robot, and benefit for the better natural human-robot interaction.

## Figures and Tables

**Table 1:** 3D object semantic segmentation experiments of our method on 10 sequences of SceneNN dataset (units: %).

	Sequence ID	011	016	030	061	078	086	096	206	223	255
Categories											
Bed		-	56.9	-	-	-	-	65.9	-	-	-
Chair		63.1	0	67.4	-	77.7	54.4	59.4	41.4	51.3	-
Sofa		-	72.1	62.9	72.5	-	-	-	65.1	-	-
Table		61.2	-	59.4	0	42.6	0	5.2	77.2	41.8	-
Books		-	-	52.9	-	53.8	45.8	20.3	-	-	-
Refrigerator		-	-	-	-	0	-	-	-	-	56.4
Television		-	-	-	-	72.2	73.6	45.1	-	-	-
Toilet		-	-	-	-	-	-	-	-	-	-
Bag		-	-	-	-	-	55.0	0	0	-	-
Average		62.2	43.0	60.7	36.3	49.3	45.8	32.7	46.0	46.6	56.4

**Table 2:** Comparative experiments on 10 sequences of SceneNN dataset (units: %).

	Sequence ID	011	016	030	061	078	086	096	206	223	255
Categories											
[31]		52.1	34.2	56.8	59.1	34.9	35.0	26.5	41.7	40.9	48.6
[27]		75.0	33.3	56.1	62.5	45.2	20.0	29.2	79.6	43.8	75.0
Single frame		45.8	26.4	48.2	19.7	35.1	30.7	18.3	33.9	27.2	34.8
Our method		62.2	43.0	60.7	36.3	49.3	45.8	32.7	46.0	46.6	56.4

**Table 3:** Ablation experiments on 10 sequences of SceneNN dataset (units: %).

	Sequence ID	011	016	030	061	078	086	096	206	223	255
Categories											
-Filter		60.5	41.4	57.4	34.8	47.5	44.3	30.1	43.5	43.8	54.2
-LCCP		55.2	40.8	55.1	33.4	46.2	42.4	28.5	43.2	41.5	52.8
-Filter-LCCP		54.6	40.2	52.9	31.1	44.7	41.6	26.4	40.2	40.7	48.7
Our method		62.2	43.0	60.7	36.3	49.3	45.8	32.7	46.0	46.6	56.4

**Table 4:** Details of experiments on 10 sequences of SceneNN dataset.

	Sequence ID	011	016	030	061	078	086	096	206	223	255
Items											
Images		3700	1300	4100	3400	7000	5900	9500	10100	4500	5400
Keyframes		370	130	410	340	700	590	950	1010	450	540
Detected objects by Mask R-CNN		361	190	4740	881	4621	1637	2267	3111	1172	1540
Object probability beyond threshold		190	140	2788	523	2407	1278	1174	1937	632	846
Manhattan frame estimation		321	260	2832	773	2239	1808	1497	3404	982	1172
Object fusion		151	124	1255	361	976	832	650	1603	446	495
Object database before refinement		21	14	204	30	185	120	147	190	80	171
Object database after refinement		8	3	75	6	53	32	35	36	15	32

**Table 5:** Runtime evaluation of our method.

Components	Time (ms)
Mask R-CNN	192.3
Object extraction and filter	160.9
LCCP	51.5
MFE	43.4
Other	66.4
Total	514.5
